# A *GSDMB* enhancer-driven HSV thymidine kinase-expressing vector for controlling occult peritoneal dissemination of gastric cancer cells

**DOI:** 10.1186/s12885-015-1436-1

**Published:** 2015-05-29

**Authors:** Norihisa Saeki, Rie Komatsuzaki, Fumiko Chiwaki, Kazuyoshi Yanagihara, Hiroki Sasaki

**Affiliations:** 1 Division of Genetics, National Cancer Center Research Institute, Tsukiji 5-1-1, Chuo-ku, Tokyo 104-0045 Japan; 2 Department of Translational Oncology, National Cancer Center Research Institute, Tsukiji 5-1-1, Chuo-ku, Tokyo 104-0045 Japan; 3 Division of Pathology, Exploratory Oncology Research & Clinical Trial Center, National Cancer Center Hospital East, Kashiwanoha 6-5-1, Kashiwa, Chiba 277-8577 Japan

**Keywords:** Stomach neoplasms, Peritoneal cavity, Genetic therapy, HSV, Thymidine kinase

## Abstract

**Background:**

Gastric cancer (GC) is one of the major malignant diseases worldwide, especially in Asia, and Japan and Korea have the highest incidence in the world. Because most of the cases that are refractory to therapies die due to peritoneal dissemination (PD) of the cancer cells, controlling PD is important for patient survival. *GSDMB* is a member of the gasdermin gene family. Because *GSDMB* is expressed in many types of cancer, including GC, it is likely that the gene contains a regulatory region that is utilized for therapy of occult PD through cancer cell-specific expression of cytotoxic genes.

**Methods:**

We performed reporter assays to identify the regulatory region for the cancer cell-specific expression. We also constructed a lentiviral therapeutic vector that expresses herpes simplex virus thymidine kinase (HSVtk) in a GC cell-specific manner, and tested it in a mouse model of PD.

**Results:**

We identified the regulatory region at +496 to +989 from the *GSDMB* transcription start site and designated it as a *GSDMB* enhancer. The lentiviral therapeutic vector suppressed proliferation of a GC cell line, 60As6, *in vitro* in the presence of ganciclovir, and intraperitoneal administration of the vector prolonged the survival term of mice that were intraperitoneally inoculated with 60As6 one week prior to the administration.

**Conclusions:**

The *GSDMB*-driven HSVtk expression vector had a therapeutic effect on the occult PD model mice. This strategy can potentially be used to treat GC patients with PD.

**Electronic supplementary material:**

The online version of this article (doi:10.1186/s12885-015-1436-1) contains supplementary material, which is available to authorized users.

## Background

Gastric cancer (GC) is one of the major malignant diseases, especially in Asia, and the second leading cause of cancer-associated deaths worldwide [1]. It is usually classified into two types (Lauren’s classification) [2], intestinal and diffuse, which are thought to reflect its pathogenesis [3]. The diffuse-type GC (DGC) is sub-classified as poorly differentiated GC (non-solid type) or undifferentiated GC in the Japanese Gastric Cancer Association classification system [4]. DGC is infiltrative and often shows aggressive invasion into the gastric wall, resulting in metastasis and the spread of GC cells into the peritoneal cavity (peritoneal dissemination, PD).

The disseminated GC cells in the peritoneal cavity give rise to peritoneal carcinomatosis (PC) [5]. PC causes gastrointestinal symptoms, such as abdominal pain, nausea and vomiting, as well as systemic symptoms such as weight loss and ascite. PC not only strongly deteriorates the quality of life of GC patients, but it is also the leading cause of death in GC [6]. With supportive care alone, the median survival of patients with PC is 3–6 months [7]. If treated with systemic chemotherapy, in the same manner as for other metastatic lesions, PC shows a poorer response to the therapy than other types of metastasis in GC, mainly because of poor distribution of the chemotherapeutic agent in the peritoneal cavity. Therefore, recent efforts have focused on innovative PC therapeutics, such combining of cytoreductive surgery, thermal therapy, and intraperitoneal chemotherapy. These combined approaches have slightly improved the prognosis of PC, although the median survival period is still less than 12 months, making it clear that there is a practical limit to the efficacy of surgical cytoreduction [8, 9]. Recent studies suggest that it is important to identify GC patients with occult PD by performing a cytologic examination of peritoneal lavage fluid, because such cases showed improved prognosis if they obtained conversion to negative cytology by extensive intraoperative peritoneal lavage followed by intraperitoneal chemotherapy [10].

The concept of “suicide gene” cancer therapy, using herpes simplex virus thymidine kinase (HSVtk), emerged in the 1980s [11]. HSVtk catalyzes the phosphorylation of the guanosine analogue ganciclovir (GCV) into a monophosphate form that is subsequently phosphorylated by cellular nucleotide kinases into highly toxic ganciclovir triphosphate [12]. Ganciclovir triphosphate blocks DNA replication, leading to cell cycle arrest and cell death [13]. Therapy involving HSVtk transfer into cancer cells, followed by GCV administration, is known as suicide gene therapy, and this technique was recently used in a phase III clinical trial on glioblastoma multiforme [12].

In this study, we developed a therapeutic vector that expresses HSVtk in cancer cells, utilizing a regulatory region of the gasdermin B gene (*GSDMB*). *GSDMB* is a member of the gasdermin (*GSDM*) family that consists of four genes, *GSDMA*, *GSDMB*, *GSDMC* and *GSDMD* [14, 15], and is expressed in proliferating cells of normal epithelium and also in many types of cancer, including esophageal, gastric, liver, colon, uterine cervix and breast cancers [14, 16–18]. *GSDMB* expression is driven by two promoters, the cellular promoter and LTR-derived promoter [19–21]. The LTR-derived promoter (LTR promoter) is active in most normal tissues, except the stomach, and in many cancer cell lines, while the cellular promoter is active in normal stomach tissue and in some cancer cell lines [20]. In this study, we identified a region in down-stream of the LTR promoter, that showed strong transcriptional activity in GC cell lines. We used this region to construct an HSVtk-expression viral vector for controlling occult PD.

## Methods

### Human tissues

Gastric cancer (GC) tissues were provided by the National Cancer Center Hospital after obtaining written informed consent from each patient, which was approved by the National Cancer Center Institutional Review Board (ID: No.17-030). Tissue specimens were immediately frozen with liquid nitrogen after surgical extraction, and stored at −80 °C until use.

### Microarray analysis

Total RNA was isolated by suspending the cells in ISOGEN lysis buffer (Nippon Gene, Toyama, Japan) followed by precipitation with isopropanol. We performed expression analyses using Human Expression Array U95A version 2 (Affymetrix, Santa Clara, CA) according to the suppliers’ protocols . The expression value (average difference: AD) of each gene was calculated using GeneChip Analysis Suite version 4.0 software (Affymetrix). Hierarchical clustering of microarray data was performed using GeneSpring (Agilent Technologies Ltd., Palo Alto, CA), Microsoft EXCEL, and Cluster & TreeView [22, 23]. All microarray data have been deposited in a MIAME compliant database, GEO (accession number; GSE47007). By Wilcoxon *u*-test (*p* < 0.05) and by showing a 2-fold change, genes expressed specifically in diffuse-type GC were selected [22].

### Cell lines and primary culture of mouse mesothelial cells

Three gastric cancer cell lines, HSC-57, derived from intestinal-type GC, and HSC-59 and HSC-60, both derived from diffuse-type GC, were established and characterized by one of the authors [24]. SNU16, derived from diffuse-type GC, was provided from the American Type Culture Collection (ATCC), Two other cell lines with efficiency in producing PD mice, 60As6 and 60As6GFP (60As6 expressing green fluorescence protein), were established by the authors from the diffuse-type GC derived HSC-60 cell line after several passages of intraperitoneal transplantation to mice [25]. CC-2511, a fibroblast cell line, was purchased from Lonza, Japan (Tokyo, Japan). All cell lines were maintained in Dulbecco’s Modified Eagle Medium. Mouse mesothelial cells were harvested by injection of 10 mL of warmed 0.25 % Trypsin/EDTA solution into the peritoneal cavity [26]. The cells were incubated for 3 days in RPMI-1640 supplemented with L-glutamine, Phenol Red and HEPES (WAKO, Tokyo, Japan). Met-5A, a human mesothelial cell line, was provided by ATCC and maintained in Medium 199 (Life Technologies, Tokyo, Japan) supplemented with 3.3 nM EGF (Life Technologies), 400 nM hydrocortison (Sigma-Aldrich, St. Louis, MO USA), 870 nM Insulin (Life Technologies) and 10 % FBS.

### RT-PCR

Total RNAs from human normal organs were purchased from BioChain, Hayward, CA. Total RNAs were extracted using an RNeasy Mini kit (QIAGEN, Tokyo, Japan). After generating first-strand cDNA from total RNA using ThermoScript RT-PCR System (Life Technologies, Tokyo Japan), PCR was performed with AccuPrime™ *Pfx* DNA Polymerase (Life Technologies) under the following cycling conditions of either 35 (LTR transcripts) or 25 cycles (others): 95 °C for 1 min; 56 °C (β-actin) or 58 °C (others) for 1 min; and 72 °C for 1 min. The following primer sets were used: for cellular promoter transcript, 5′-CTTCCTGAGATTCAGAGGCC-3′ and 5′-CCAGAATTTGAAACTCAGCC-3′; for LTR promoter-derived transcripts, 5′-TTCAGTTGCTTCAGGCCATC-3′ and 5′-CCAGAATTTGAAACTCAGCC-3′; for the 3′ side of *GSDMB*, 5′-ATTCTGGACTTCCTGGATGC-3′ and 5′-ATGTATGAAATCCAGGCTGG-3′; for *MYH11*, 5′- CAGTGACGATGAGAAGTTCC-3′ and 5′- CGCAGAAGAGGCCAGAGTAC; and for *β-actin*, 5′-TCATCACCATTGGCAATGAG-3′ and 5′-CACTGTGTTGGCGTACAGGT-3′.

### Reporter Assay

A genomic fragment, from −1080 to +1053 of *GSDMB* and containing the LTR promoter, was amplified by PCR using LA Taq Hot Start DNA polymerase (Takara) in 35 cycles of 96 °C for 30 s and 68 °C for 2 min, using primer sets: 5′-CTTCCTGAGATTCAGAGGCC-3′ and 5′-CTCGAGTTCACTGTGTTAGCCAGG-3′, and inserted into a pGL3 basic vector (Promega, Madison, WI). It was truncated using the restriction sites: *Nhe* I and *Eco*R I to generate the −1035 to +1053 fragment; *Kpn*I and *Eco*R I for −426 to +1053; *Nhe* I and *Afl* II for −61 to +1053; *Nhe* I and *Eco*81 I for +129 to +1053; and *Nhe* I and *Stu* I for +496 to +1053. The +496 to +1053 reporter construct was further truncated with restriction enzymes: *Nhe* I and *Swa* I for +757 to +1053; *Nhe* I and *Pvu* II for +860 to +1053; *Nhe* I and *Bst*X I for +989 to +1053; *Xho* I and *Bst*X I for +496 to +989; *Xho* I and *Pvu* II for +496 to +860; and *Xho* I and *Swa* I for +496 to +757. For further truncation of the +496 to +989 fragment, PCR was performed with the fragment as a template using Ex Taq DNA polymerase (Takara) in 35 cycles of 95 °C for 1 min, 58 °C for 1 min, and 72 °C for 1 min, using the following primer sets: for +562 to +989, 5′-GCTAGCTGTGGGATTTGTACACATCC-3′ and 5′- AGATCTCGACTGGGATTACAGG-3′; and for +649 to +989, 5′-GCTAGCTTTATTTCCACTGGAAACCG-3′ and 5′-AGATCTCGACTGGGATTACAGG-3′. After amplification, fragments were inserted into pGL4.12[*luc2CP*] vector (Promega). The −1 kb upstream regions of *CXCR4* and *CXCR7* were prepared by genomic PCR using MightyAmp DNA polymerase (Takara) in 35 cycles of 98 °C for 10 s, 62 °C for 15 s, and 68 °C for 2 min, using the following primer sets: for *CXCR4*, 5′-GCTAGCGCGCCCACTGCAAACCTCAG-3′ and 5′-CTTAAGTCACTTTGCTACCTGCTGC-3′; and for *CXCR7*, 5′-GCTAGCCGGAGGCCCCCGGAGAGCAG-3′ and 5′-CTTAAGTTTGCAACAACTGTGAGC-3′. These fragments were inserted into the pGL4.12[*luc2CP*] vector. One microgram of each construct and the Renilla luciferase control reporter vector (pRL-SV40 vector, Promega) were co-transfected into 1 × 10^5^ cells using SuperFect Transfection Reagent (QIAGEN). The luciferase assay was performed 24 h after the reporter introduction, using a Dual-Luciferase Reporter Assay System (Promega). The assay was carried out in triplicate.

### *GSDMB* enhancer-HSVtk lentivirus vector

A pMFG-HSVtk vector was provided by RIKEN BRC through the National Bio-Resource Project of the MEXT, Japan, by courtesy of Dr. Hirofumi Hamada, and an HSVtk cDNA was excised from it as an *Nco* I-*Bam*H I fragment. To construct the *GSDMB* enhancer-HSVtk lentivirus vector, first the +496 to +989 fragment (*GSDMB* enhancer) was inserted into pcDNA3.1 (+) (Life Technologies) between *Nhe* I and *Hind* III sites, and then HSVtk cDNA was inserted into the vector at a *Bam*H I site in the forward (for sense-strand expression) or reverse (for antisense-strand expression) direction. Next, *GSDMB* enhancer-HSVtk sense and *GSDMB* enhancer-HSVtk antisense fragments were excised from the plasmid vectors as *Nhe* I-*Not* I fragments and inserted into pLVSIN-CMV neo vectors between the *Xba* I and *Not* I sites. Finally, a CMV promoter was removed from the lentiviral constructs. To generate viral particles containing the vectors, the constructs were introduced into Lenti-X™ 293 T Cells (Takara) using Lenti-X™ HTX Packaging System (Takara). After 72 h’-incubation, the medium was collected and the viral titer (cfu/mL) was determined by transduction into HT-1080 cells in the presence of polybrene (5 μg/mL in culture medium, Sigma-Aldrich). The particles were applied to Met-5A and 60As6 (1 × 10^5^ cells per dish, in triplicate) *in vitro* in the presence of polybrene (5 μg /mL), and the cells were incubated in medium containing Gancicrovir (GCV, 5 μg/mL, WAKO) for 5 days for cell growth assays. The assays were performed in triplicate and *P*-value of Student’s *t*-test between the cultured cells with (+) and without (−) GCV was calculated.

### Treatment of PD mouse model with *GSDMB* enhancer-HSVtk vectors

We previously reported a mouse PD model (PD mice) that was produced by intraperitoneal injection of 60As6 cells [25]. 60As6GFP cells (1 × 10^6^ cells per mouse) were injected into the peritoneal cavity of 18 mice (6 week-old mice of CB17/Icr-Prkdc < scid>/CrlCrlj Genotype: scid/scid, Charles River, Yokohama Japan) at day 1. The mice were divided into two groups; one group was injected with the antisense expression vector, and the other group was injected with the sense vector; both groups then were intraperitoneally injected with 2 mL of PBS solution containing viral particle (5 × 10^5^ cfu) and Ganciclovir (2 mg) at 8, 10 and 12 day. The mean survival time of each group and the *P*-value of Student’s *t*-test between the two groups were calculated. The study was approved by the National Cancer Center Committee on Animal Experiments.

## Results

### Identification of an enhancer region in *GSDMB*, which drives gene expression in GC cells

To identify the promoter/enhancer regions that would be effective in the development of a therapeutic vector for peritoneal dissemination (PD), we first searched for genes more frequently expressed in diffuse-type GC than in intestinal-type GC using comparative gene expression analysis between 12 primary diffuse-types and 18 intestinal-types, because PD is more frequently seen in diffuse-type GC than in the intestinal-type [22]. We noticed that four of ten Affymetrix GeneChip probe sets showing the highest fold-change for gene expression in diffuse-type GC compared to intestinal-type were probe sets for *MYH11* (myosin, heavy chain 11, smooth muscle gene, Additional file 1: Table S1). After confirming that the gene is not expressed in the immortalized human mesothelial cell line MeT-5A (data not shown), we selected *MYH11* as a strong candidate for the gene whose promoter enables diffuse-type GC specific expression of HSVtk. However, the gene is not expressed in 60As6 cells that were used for making PD model mice (Additional file 2: Figure S1). It is likely that *MYH11* is expressed in cancer-associated fibroblasts which are especially abundant in diffuse-type GC tissues. Next, going out of the microarray data analysis, we shifted our attention to upstream regions of *CXCR4* (chemokine (C-X-C motif) receptor 4 gene) and *CXCR7* (chemokine (C-X-C motif) receptor 7 gene), as both are expressed in many types of cancer and have an important role in metastasis [27]. However, using reporter assays, we found that the upstream regions of these genes were transcriptionally active in both the MeT-5A and the 60As6 cells (Additional file 2: Figure S2), implying that the regions drive the expression of HSVtk in human mesothelial cells *in vivo*. Finally, we focused on the *GSDMB* gene, as our previous study indicated that it is strongly expressed in GC tissues and cell lines [14].

**Fig. 1:**
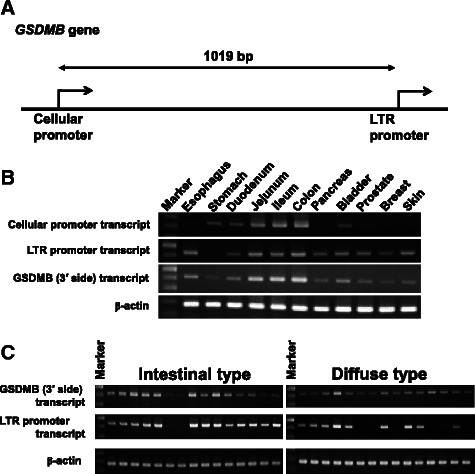
*GSDMB* gene is transcribed by the Cellular and LTR promoters. (**a**) A schematic illustration of the two promoters. (**b**) Expression of two transcripts, one by cellular promoter and the other by LTR, in human normal tissues (RT-PCR). Four variants of human GSDMB transcript are registered in GenBank; variant 1 (NM_001042471), variant 2 (NM_018530), variant 3 (NM_001165958) and variant 4 (NM_001165959). Transcription of variants 1, 3 and 4 is driven by the cellular promoter and that of variant 2 is by the LTR promoter. The 3′ side of the *GSDMB* transcripts is common to each. (**c**) Expression of LTR transcripts in gastric cancer tissues, 15 intestinal-type and 15 diffuse-type samples (RT-PCR on surgical specimens)

*GSDMB* is transcribed by two promoters, cellular and LTR promoters (Fig. 1a), and the latter is mainly used in normal tissues and in cancer cell lines [19–21]. We confirmed these findings by performing RT-PCR analyses on RNA from several types of normal tissues (Fig. 1b). RT-PCR on GC surgical specimens demonstrated that the LTR promoter was used in 14 of 15 intestinal-type GCs and in 11 of 15 diffuse-type GCs (Fig. 1c).

**Fig. 2:**
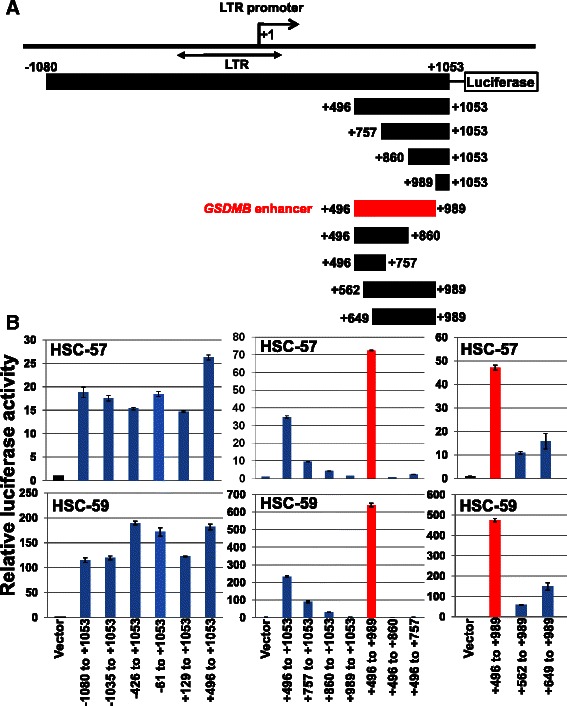
Identification of *GSDMB* enhancer. (**a**) A schematic illustration showing reporter constructs used in the luciferase assays. Long terminal repeat (LTR) element of human endogeneous retrovirus is shown by a double-headed arrow. The position is from the transcription start site for the transcript of the LTR promoter. (**b**) Luciferase assays using two gastric cancer cell lines, HSC-57 and HSC-59, revealed a region with strong transcriptional activity, spanning from +496 to +989, which was designated as *GSDMB* enhancer. Vector, empty reporter vector, Bar, standard deviation

To identify a region critical for the transcriptional activity in GC cells, a DNA fragment spanning −1080 to +1053 bp, the position from a transcription start site for the LTR promoter, was isolated (Fig. 2a). The reporter assays on truncated DNA fragments using two GC cell lines, HSC-57 and HSC-59, indicated that a +496 to +989 region had strong transcriptional activity, even stronger than that of the original −1080 to +1053 fragment, and that further truncation of the +496 to +989 fragment resulted in significant reduction of the transcriptional activity (Fig. 2b). The region corresponding to this fragment with strong transcriptional activity was named *GSDMB* enhancer.

### Construction of a *GSDMB* enhancer-driven HSVtk lentivirus vector

**Fig. 3:**
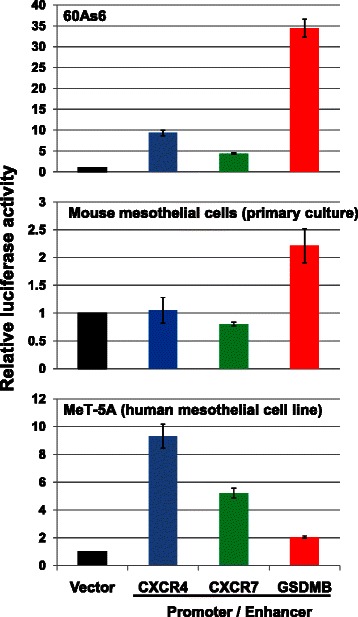
*GSDMB* enhancer has strong transcriptional activity in a 60As6 cell line. Luciferase assays with three types of cultured cells: 60As6 cells that were used for making peritoneal dissemination (PD) model mice in this study, primary culture cells of mouse peritoneal mesothelial cells and established human mesotherial cell line Met-5A. Bar, standard deviation

We previously reported a mouse PD model (PD mice) that was produced by intraperitoneal injection of 60As6 cells [25]; in this study, we developed a viral therapeutic vector for the treatment of PD mice. For examining the strength of the transcriptional activity of *GSDMB* enhancer in 60As6, reporter assays were performed, using the reporter construct for the upstream regions of *CXCR4* and *CXCR7* for comparison. The *GSDMB* enhancer showed stronger transcriptional activity in 60As6 cells than the *CXCR4* or the *CXCR7* upstream regions, and, importantly, the *GSDMB* enhancer had very weak transcriptional activity in mouse peritoneal mesothelial cells and in Met-5A, a human mesothelial cell line (Fig. 3). This result suggests that the *GSDMB* enhancer enables HSVtk expression almost exclusively in 60As6 but not in mesothelial cells of the peritoneal cavity of the PD mice, and probably not in human peritoneal mesothelium.

**Fig. 4:**
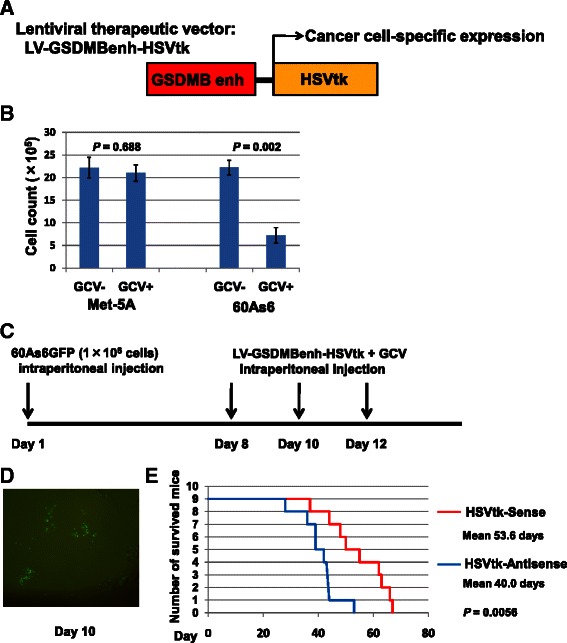
HSVtk/GCV therapy using the *GSDMB* enhancer-driven lentivirus vector improved the survival rate of PD mice. (**a**) A lentiviral therapeutic vector for *GSDMB* enhancer (Enh)-driven expression of herpes simplex virus thymidine kinase (HSVtk). (**b**) Cell proliferation assays on 60As6 and Met-5A transduced with the therapeutic vector, performed by incubation in the medium with (+)/without (−) ganciclovir (GCV). (**c**) A regimen of HSVtk/GCV therapy for PD mice. Bar, standard deviation, *P*, *P* -value of Student’s *t*-test between the cultured cells with (+) and without (−) GCV. (**d**) Microscopic observation exhibited a small population of 60As6GFP cells (green fluorescence) implanted into mouse peritoneum at day 10. (**e**) Number of survived mice after HSVtk/GCV therapy with the sense-strand expressing vector (red) and with an antisense-strand expressing vector as reference (blue). Mean survival time of each group is shown at the right side with *P*- value of Student’s *t*-test between the two groups

Next, we examined the effect of the HSVtk/GCV therapy using the *GSDMB* enhancer-driven HSVtk lentivirus vector on 60As6 *in vitro* (Fig. 4a). The number of 60As6 cells transduced with the lentivirus vector was significantly reduced when incubated in medium supplemented with GCV; on the other hand, the same HSVtk/GCV treatment had no effect on the cell number of Met-5A (Fig. 4b).

### HSVtk/GCV therapy of occult PD mice

We applied HSVtk/GCV therapy to PD mice. In this therapeutic assay, we prepared two types of the *GSDMB* enhancer-driven lentivirus vector: one vector expressed the sense-strand of HSVtk cDNA and was used for the treatment of PD mice, whereas the other vector expressed the antisense-strand and was used as the control. The therapy was started seven days after intraperitoneal inoculation of 60As6 cells expressing green fluorescence protein (60As6GFP). This regimen was designed for treatment of occult PD model in which 60As6GFP cells were diffusely engrafted into the peritoneal cavity (Figs. 4c, d). After three doses of treatment, at day 36, none of the nine mice treated with HSVtk sense-expression vector had died, while two of the nine reference mice had already died. None of the nine reference mice were alive at day 57, i.e., eight weeks after injection of 60As6GFP cells; however, four of nine therapeutic vector-treated mice were still alive (Fig. 4e). This result suggests that the therapy can improve the prognosis of occult PD mice.

## Discussion

### The *GSDMB* enhancer drives gene expression in GC cells

Previously we reported that *GSDMB* is expressed in all GC tissues and cell lines examined [14], and in this study we demonstrated that the LTR promoter drives *GSDMB* expression in 25 of 30 GC specimens (Fig. 1c). The transcriptional activity of the LTR region (Fig. 2a) was previously demonstrated by reporter assays in non-GC cell lines [20, 21]. However, we found a distinct region with strong transcriptional activity in the downstream of the LTR region, and designated it as *GSDMB* enhancer. In addition to the two GC cell lines, HSC-57 and HSC-59, the transcriptional activity of this region was detected by reporter assays in other GC cell lines, including MKN74 (relative luciferase activity was approximately 1.9), HSC-60 (29.4), HSC-42 (2.5) and HSC-44 (4.6), but not in HSC-58 or MKN28 (data not shown) [14]. Thus, the *GSDMB* enhancer does not drive gene expression in some GC cells.

Truncation of a region spanning +496 to +562 significantly reduced the transcriptional activity of the *GSDMB* enhancer (Fig. 2b, +562 to +989). In the +496 to +562 region, we found consensus-binding sites of several transcription factors, including GATA2, GATA3, GATA4, YY1, SOX5, SOX9, SOX10 and NFY-A, and base-substitution in any of these consensus sequences did not affect the transcriptional activity of the enhancer (data not shown). The transcription factor that interacts with the enhancer and contributes to its transcriptional activity has not yet been identified.

### Application of the therapeutic lentivirus vector to treatment of human occult PD

Curative therapy has not been established for PD. GC patients with macroscopic PD have poor prognoses, with a median overall survival of 3–6 months. Those with only microscopic PD also have a poor prognosis; their 5-year survival rate is 0-18 % [28]. Therefore, it is important to detect occult PD by cytologic examination of peritoneal lavage fluid and completely eradicate cancer cells in the peritoneal cavity. Meta-analyses by Cabalag *et al*. indicated that extensive intraperitoneal lavage (EIPL, physiological saline 1 litter/dose, 10 times) and intraoperative intraperitoneal chemotherapy (IIPC) with cisplatin significantly improved 5-year overall survival to more than 40 % [28]. The results of our study suggest that HSVtk/GCV therapy using the lentivirus vector improves the prognosis of patients independently, and we assume it will be used as a consolidation therapy. Solid tumors with diffuse growth are composed of many myofibroblasts and few vessels (e.g., diffuse-type GCs, pancreatic cancers and scirrhous type of breast cancer). Depending on the conditions of the microenvironment, such as nutrient deficiency, these tumors show a high prevalence of rarely-proliferative tumor cells. Thus, diffuse-type GC cells disseminated in the peritoneal cavity may consist of a population that can resist the cytotoxic effect of cisplatin. The lentivirus therapeutic vector can introduce HSVtk into both proliferating and non-proliferating cells. Moreover, the *GSDMB* enhancer enables GC cell-specific HSVtk expression. This restricted expression minimizes mesothelial cell damage, implying that the gene therapy can be performed using doses high enough to completely eradicate GC cells, even those resistant to cisplatin, in occult PD. It is likely that combination therapy, EIPL and IIPC, followed by HSVtk/GCV therapy using the lentivirus vector, will improve the prognosis of occult PD more significantly than the EIPL and IIPC combination therapy alone. We believe that this regimen is worthy of being placed on clinical trials. Although it appears that the *GSDMB* enhancer does not work in some GC cells, further studies aiming at identifying additional GC-specific enhancers, will resolve this problem.

## Conclusions

The *GSDMB*-driven HSVtk expression vector had a therapeutic effect on the occult PD model mice. This strategy can potentially be used to prevent GC patients from contracting PD and also used to treat GC patients with PD.

## Additional files

Additional file 1: Table S1. Top ten probe sets showing expression specific to diffuse-type gastric cancer.
Additional file 2: Figure S1. *MYH11* is not expressed in gastric cancer cell lines. A promoter region of both *CXCR4* and *CXCR7* genes shows a transcriptional activity in both 60As6 and MeT-5A cells.

## References

[1] Brenner H, Rothenbacher D, Arndt V, Verma M (2009). Epidemiology of gastric cancer. Methods of molecular biology, cancer epidemiology, vol. 472.

[2] Lauren P (1965). The two histological main types of gastric carcinoma: diffuse and so-called intestinal-type carcinoma. An attempt at a histoclinical classification. Acta Pathol Microbiol Scand.

[3] Yasui W, Sentani K, Motoshita J, Nakayama H (2006). Molecular pathobiology of gastric cancer. Scand J Surg.

[4] Japanese Gastric Cancer Association (1998). Japanese classification of gastric carcinoma. - 2nd English edition. Gastric Cancer.

[5] Rosai J (2004). Gastrointesitinal tract – stomach. Rosai and Ackerman’s surgical pathology.

[6] Maruyama K, Kaminishi M, Hayashi K, Isobe Y, Honda I, Katai H (2006). Gastric cancer treated in 1991 in Japan: data analysis of nationwide registry. Gastric Cancer.

[7] Davies JM, O’Neil B (2009). Peritoneal carcinomatosis of gastrointestinal origin: natural history and treatment options. Expert Opin Investg Drugs.

[8] Ströhlein MA, Bulian DR, Heiss MM (2011). Clinical efficacy of cytoreductive surgery and hyperthermic chemotherapy in peritoneal carcinomatosis from gastric cancer. Expert Rev Anticancer Ther.

[9] Brücher BL, Piso P, Verwaal V, Esquivel J, Derraco M, Yonemura Y (2012). Peritoneal carcinomatosis: cytoreductive surgery and HIPEC–overview and basics. Cancer Invest.

[10] De Andrade JP, Mezhir JJ (2014). The critical role of peritoneal cytology in the staging of gastric cancer: An evidence-based review. J Surg Oncol.

[11] Moolten FL (1986). Tumor chemosensitivity conferred by inserted herpes thymidine kinase genes: paradigm for a prospective cancer control strategy. Cancer Res.

[12] Wirth T, Parker N, Ylä-Herttuala S (2013). History of gene therapy. Gene.

[13] Wei SJ, Chao Y, Hung YM, Lin WC, Yang DM, Shih YL (1998). S- and G2-phase cell cycle arrests and apoptosis induced by ganciclovir in murine melanoma cells transduced with herpes simplex virus thymidine kinase. Exp Cell Res.

[14] Saeki N, Usui T, Aoyagi K, Kim DH, Sato M, Mabuchi T (2009). Distinctive expression and function of four *GSDM* family genes (*GSDMA-D*) in normal and malignant upper gastrointestinal epithelium. Genes Chromosomes Cancers.

[15] Saeki N, Sasaki H, Carrasco J, Mota M (2012). Gasdermin superfamily: a novel gene family functioning in epithelial cells. Endothelium and Epithelium: composition, functions and pathology.

[16] Carl-McGrath S, Schneider-Stock R, Ebert M, Röcken C (2008). Differential expression and localisation of gasdermin-like (GSDML), a novel member of the cancer-associated GSDMDC protein family, in neoplastic and non-neoplastic gastric, hepatic, and colon tissues. Pathology.

[17] Sun Q, Yang J, Xing G, Sun Q, Zhang L, He F (2008). Expression of GSDML associates with tumor progression in uterine cervix cancer. Transl Oncol.

[18] Hergueta-Redondo M, Sarrió D, Molina-Crespo Á, Megias D, Mota A, Rojo-Sebastian A (2014). Gasdermin-B promotes invasion and metastasis in breast cancer cells. PLoS One.

[19] Komiyama H, Aoki A, Tanaka S, Maekawa H, Kato Y, Wada R (2010). Alu-derived cis-element regulates tumorigenesis-dependent gastric expression of GASDERMIN B (GSDMB). Genes Genet Syst.

[20] Sin HS, Huh JW, Kim DS, Kang DW, Min DS, Kim TH (2006). Transcriptional control of the HERV-H LTR element of the GSDML gene in human tissues and cancer cells. Arch Virol.

[21] Huh JW, Kim DS, Kang DW, Ha HS, Ahn K, Noh YN (2008). Transcriptional regulation of GSDML gene by antisense-oriented HERV-H LTR element. Arch Virol.

[22] Suzuki M, Chiwak F, Sawada Y, Ashikawa M, Aoyagi K, Fujita T, et al. Peripheral opioid antagonist enhances the effect of anti-tumor drug by blocking a cell growth-suppressive pathway in vivo. PLoS One. (in press) 10.1371/journal.pone.0123407 PMC4390307 25853862

[23] Aoyagi K, Minashi K, Igaki H, Tachimori Y, Nishimura T, Hokamura N (2011). Artificially induced epithelial-mesenchymal transition in surgical subjects: its implications in clinical and basic cancer research. PLoS One.

[24] Yanagihara K, Seyama T, Tsumuraya M, Kamada N, Yokoro K (1991). Establishment and characterization of human signet ring cell gastric carcinoma cell lines with amplification of the c-myc oncogene. Cancer Res.

[25] Fujita T, Yanagihara K, Takeshita F, Aoyagi K, Nishimura T, Takigahira M (2013). Intraperitoneal delivery of a small interfering RNA targeting NEDD1 prolongs the survival of scirrhous gastric cancer model mice. Cancer Sci.

[26] Bot J, Whitaker D, Vivian J, Lake R, Yao V, McCauley R (2003). Culturing mouse peritoneal mesothelial cells. Pathol Res Pract.

[27] Sun X, Cheng G, Hao M, Zheng J, Zhou X, Zhang J (2010). CXCL12/CXCR4/CXCR7 chemokine axis and cancer progression. Cancer Metastasis Rev.

[28] Cabalag CS, Chan ST, Kaneko Y, Duong CP (2015). A systematic review and meta-analysis of gastric cancer treatment in patients with positive peritoneal cytology. Gastric Cancer.

