# Supplementary material for: A GSDMB enhancer-driven HSV thymidine kinase-expressing vector for controlling occult peritoneal dissemination of gastric cancer cells
Source: BMC Cancer. 2015 May 29;15:439. doi: 10.1186/s12885-015-1436-1 (PMC4446855; doi:10.1186/s12885-015-1436-1)
Supplement: Additional file 2: Figure S1. — MYH11 is not expressed in gastric cancer cell lines. A promoter region of both CXCR4 and CXCR7 genes shows a transcriptional activity in both 60As6 and MeT-5A cells. [file 12885_2015_1436_MOESM2_ESM.pdf]

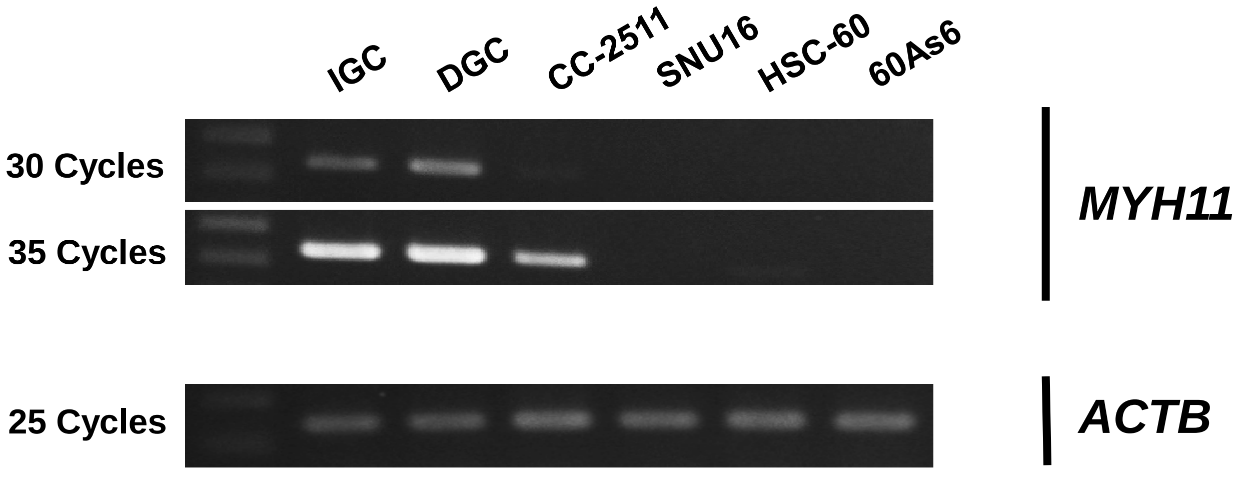

Additional Figure 1 *MYH11* is not expressed in gastric cancer cell lines. Results from RT-PCR indicating that *MYH11* is expressed in a fibroblast cell line (CC-2511) but not in gastric cancer cell lines (SNU16, HSC-60 and 60As6). The *MYH11* transcripts detected in gastric cancer tissues (DGC and IGC) may be derived from transcripts in cancer-associated fibroblast in the tissues. RNA samples of DGC and IGC are a mixture of RNA from five tissues of diffuse- and intestinal-type gastric cancers, respectively.

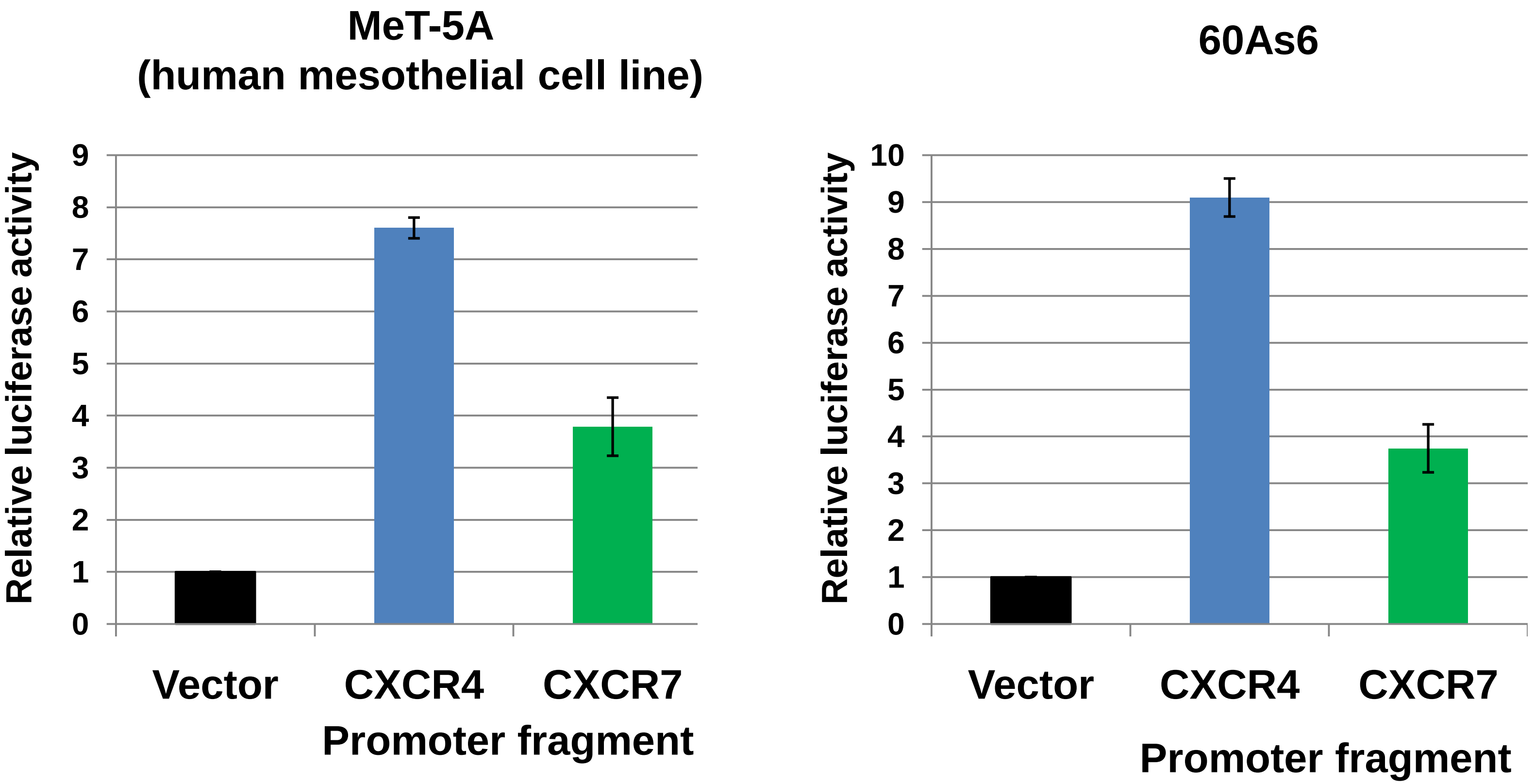

Additional Figure 2 A promoter region of both *CXCR4* and *CXCR7* genes shows a transcriptional activity in both 60As6 and MeT-5A cells. Luciferase reporter assays on the promoter region of the tow genes were performed, which renealed that they have relatively strong transcriptional activities in MeT-5A, a human mesothelial cell line. An empty vector was used as a reference. Bars, standard deviation.
